# Comparison of hemostatic effect and safety between epinephrine and tramazoline during nasotracheal intubation: a double-blind randomized trial

**DOI:** 10.1186/s12871-021-01454-y

**Published:** 2021-09-30

**Authors:** Aiji Sato-Boku, Yoshiki Sento, Yuji Kamimura, Eisuke Kako, Masahiro Okuda, Naoko Tachi, Yoko Okumura, Mayumi Hashimoto, Hiroshi Hoshijima, Fumihito Suzuki, Kazuya Sobue

**Affiliations:** 1 Department of Anesthesiology, Aichi Gakuin University School of Dentistry, 2-11 Suemori-dori, Chikusaku, Nagoya, 464-8651 Japan; 2 Department of Anesthesiology and Intensive Care Medicine, Nagoya City University Graduate School of Medical Sciences, 1 Kawasumi, Mizuho-cho, Mizuho-ku, Nagoya, Aichi Japan; 3 Division of Dento-oral Anesthesiology, Tohoku University Graduate School of Dentistry, Seiryomachi 4-1, Aoba, Sendai, Miyagi Japan; 4 Department of Dentisitry, National Hospital Organization Akita National Hospital, 84-40, Azaidono, Uchimigawa, Iwaki, Yurihonjyo, Akita, Japan

**Keywords:** Nasotracheal intubation, Epinephrine, Tramazoline, Nasal bleeding

## Abstract

**Background:**

Nasal bleeding is the most common complication during nasotracheal intubation (NTI). To reduce nasal bleeding, the nasal mucosa is treated with vasoconstrictors (epinephrine [E] or tramazoline [T]) prior to NTI. This study aimed to determine whether E or T is more effective and safe for reducing nasal bleeding during NTI.

**Methods:**

This study was preregistered on UMIN-CTR after being approved by the IRB of the School of Dentistry at Aichi Gakuin University. Written consent was received from all the patients. Total 206 patients aged 20–70 years and classified as 1–2 on American Society of Anesthesiologists-physical status were scheduled to undergo general anesthesia with NTI. At last, 197 patients were randomly divided into two groups and treated with either E (n = 99; 3 patients were discontinued) or T (n = 98; 2 patient were discontinued). After induction of general anesthesia, each patient’s nasal mucosa was treated using either E or T. The E used in this study was BOSMIN® SOLUTION 0.1% (Daiichi-Sankyo Co., Ltd., Tokyo), and the T used in this study was TRAMAZOLIN Nasal Solution 0.118% AFP, (Alfresa Pharma Corporation, Osaka). E was diluted five times according to the package insert (final concentration of E = 0.02%), and T was used in its original solution. After 2 min, NTI was performed via the right nostril. Primary outcome were the presence of nasal bleeding (if bleeding was recognized at the posterior pharyngeal wall via nasal cavity during intubation, it was defined as bleeding) and the degree of bleeding (classified as none, mild, moderate, or severe). Secondary outcomes were arrhythmia, and hemodynamic (mean atrial pressure and heart rate) changes associated with vasoconstrictors.

**Results:**

The presence of bleeding was comparable in both groups (12.5%, E; 14.5%, T; *P* = 0.63). No significant difference between the groups regarding the degree of bleeding (*P* = 0.78) was observed, with most patients having no bleeding (n = 84, E; n = 82, T). No severe bleeding and no arrhythmias induced by vasoconstrictor were observed in the two groups.

**Conclusions:**

Nasal treatment with E or T shows no difference in nasal bleeding during NTI. Although no arrhythmia associated with E was observed in this study, it has been reported in literature. Therefore, as frequency and degree of nasal bleeding were comparable, nasal treatment with T could reduce the risk of NTI.

**Trial registration:**

UMIN-CTR (Registration No. UMIN000037907). Registered (05/09/2019).

## Background

Nasotracheal intubation (NTI) is frequently necessary during dental, maxillofacial, and oropharyngeal surgeries. Although this method is very useful in operations wherein the operative field and airway are congruent, some complications associated with NTI include nasal bleeding [1, 2], bacteremia [3], retropharyngeal perforation [4], and partial or complete obstruction of the tube [5, 6] has been reported. Several effective preventive measures against nasal bleeding, retropharyngeal perforation, and bacteremia have been reported [7–10]. Previous studies [11–13] have compared vasoconstriction effect of epinephrine (E) and xylometazoline during NTI; however, xylometazoline is not available in authors’ country. The nasal mucosa is usually treated with vasoconstrictors, such as E or tramazoline (T), prior to NTI in authors’ country. While xylometazoline is an imidazole derivative designed to mimic the molecular shape of epinephrine and binds with alpha 1 and 2 adrenergic receptors and can induce vasoconstriction [14], T stimulates alpha 2 receptors and constricts local blood vessels in the nasal cavity, eliminating hyperemia and swelling of the nasal mucosa [15]. Thus the kind of vasoconstrictors are still debatable during NTI. T has been used for treatment since 1960 and E even before that. Considering their significant use in medical procedures for >60 years, their efficacy and side effects are well known. Although arrhythmia and cardiac arrest have been reported in literature for nasal treatments performed with E and oxymetazoline [16, 17] so far, no previous reports compared the vasoconstriction effects and safety of vasoconstrictors during NTI. This study aimed to determine whether E or T is more effective and safe for reducing nasal bleeding during NTI. To the best of our knowledge, this is the first study to compare E and T.

## Methods

### Ethics approval and consent to participate

This study was performed in accordance with the ethical standards of the Declaration of Helsinki (1964) and its subsequent amendments. This study was adhered to CONSORT guidelines. This study was approved by the Ethics Committee at the School of Dentistry, Aichi Gakuin University (Approval No. 566), and written informed consent was obtained from all patients participating in the trial. Prior to patient enrollment, the trial was registered as a clinical trial at UMIN-CTR (Registration No. UMIN000037907, Date of first registration: 05/09/2019). The first patient was recruited and registered on 4th October, 2019.

### Study design and population

**Fig. 1:**
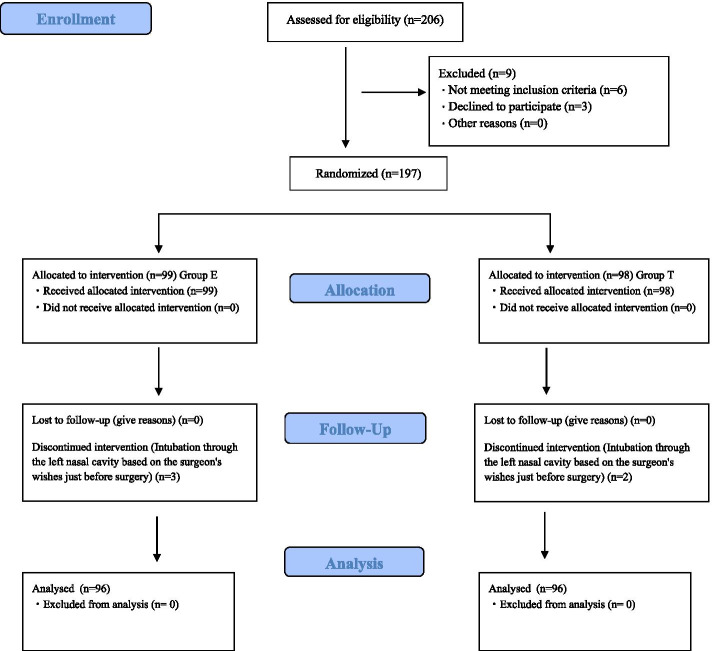
CONSORT diagram recommended description of patient flow through the study recruitment

We conducted a prospective, double-blind randomized study, enrolling 206 patients aged 20–70 years and classified into classes 1 and 2 as per American Society of Anesthesiologists-physical status that were scheduled to undergo general anesthesia with NTI for oral and maxillofacial surgery. Among them, patients with a narrowed nasal mucosa observed during preoperative CT test (n = 3), patients with obstructed nasal breathing (n = 0), patients with hypertension and using antihypertensive drugs (n = 3), patients with abnormal coagulation in preoperative blood test (n = 0), and patients undergoing antithrombotic therapy (n = 0) were excluded. In addition, patients who did not give consent (n = 3) were excluded from the study. Therefore, the final study population included 197 patients who were randomly divided into two groups: the group treated with E (n = 99) and the group treated with T (n = 98). A researcher who was not the anesthesia provider in charge conducted the randomization in accordance with a computer-generated random number (Fig. 1).

### Anesthesia and intubation methods

**Fig. 2:**
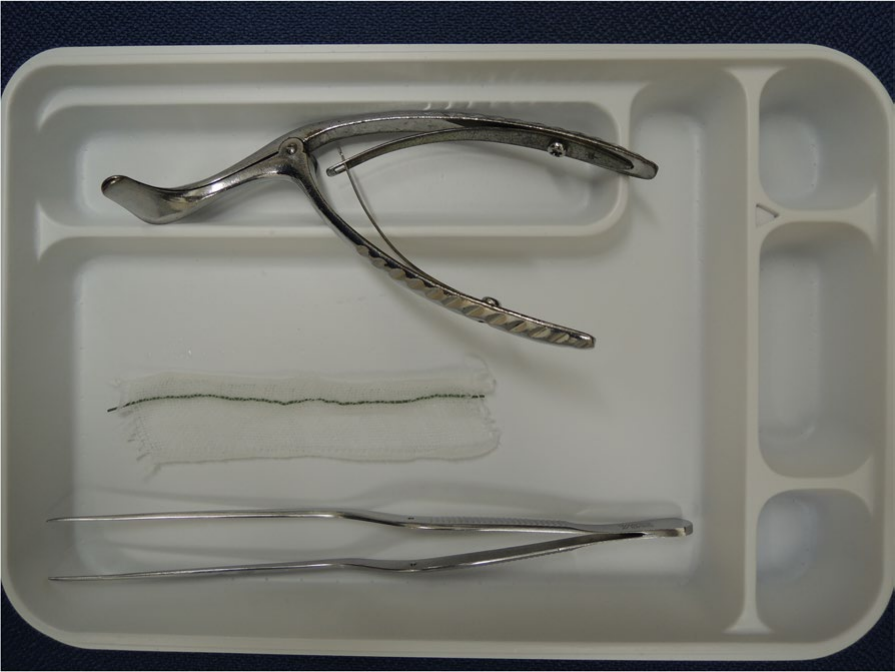
A 10 cm x 3 cm gauze in 2 ml of pre-specified chemical solution (E or T) , a rhino scope and a louche

The same method of anesthesia was employed for all patients. No premedication was administered. After a patient walked independently to the operating theater, the standard vital signs monitors (electrocardiogram, blood pressure, and oxygen saturation) were inspected. Anesthesia was induced using propofol (4 μg/ml target control infusion), remifentanil (0.2 μg/kg/min), and fentanyl (100 μg) with rocuronium (0.6 mg/kg) used as a neuromuscular blocking agent. Until the effects of the neuromuscular blocking agent became apparent, mask ventilation was applied in all patients using 100% oxygen with propofol and remifentanil. While mask ventilation was being performed, the patients’ nasal mucosa and inferior nasal passages were adequately disinfected with benzalkonium [10] spray (ZALKONIN® SOLUTION 0.025, Kenei Phamaceutical Co., Ltd., Osaka). During disinfection, swabbing was not done to avoid nasal bleeding. Next, the anesthesia provider in charge soaked a 10 cm × 3 cm gauze in 2 ml of chemical solution (E or T) which was randomly pre-specified by researcher. Next, the gauze was inserted through the nasal mucosa into the lower nasal passage using a rhino scope and louche (Fig. 2). After 2 min, the gauze was removed and NTI was performed in the right nostril [7]. The E used in this study was BOSMIN® SOLUTION 0.1% (Daiichi-Sankyo Co., Ltd., Tokyo), and the T used in this study was TRAMAZOLIN Nasal Solution 0.118%「AFP」, (Alfresa Pharma Corporation, Osaka). E was diluted five times according to the package insert (final concentration of E = 0.02%), and T was used in its original solution. The intubation tube used in this study was Polar™ Preformed Tracheal Tube (Smith Medical Japan Ltd., Tokyo), and the tube size was ID 7.0 for males and ID 6.5 for females.

### Measurements

The primary outcomes were the presence or absence of nasal bleeding observed at the posterior pharyngeal wall during intubation and the degree of bleeding. The assessment was done by the anesthesia provider in charge who was not informed in advance whether the chemical solution was E or T. The degree of bleeding was graded on four levels (none, mild, moderate, and severe), determined as follows: none—no bleeding on the posterior wall of the pharynx at the time of intubation and 10 min after intubation, and no bleeding from the nasal cavity at extubation; mild—bleeding in the posterior wall of the pharynx at intubation, no bleeding in the posterior wall of the pharynx 10 min after intubation, and no bleeding from the nasal cavity at extubation; moderate—bleeding on the posterior wall of the pharynx at the time of intubation and 10 min after intubation, but no bleeding from the nasal cavity at extubation; severe—bleeding on the posterior wall of the pharynx at the time of intubation and 10 min after intubation, and bleeding from the nasal cavity at extubation. Assessment of bleeding in the posterior wall of the pharynx at the time of intubation and 10 min after intubation was confirmed by direct laryngoscopy. The secondary outcome was presence or absence of arrhythmia associated with vasoconstrictors detected through an electrocardiography. In addition, mean arterial pressure (MAP) and heart rate (HR) was measured before and after the use of vasoconstrictors. The years of experience of the intubator was also recorded.

### Statistical analysis

Based on our estimation, a minimum sample of 178 patients (n = 89 per group), where the effect size, minimal significance (α) and statistical power (1 − β) were set at 0.22, 0.05, and 0.80 respectively. The effect size was calculated on the basis of the statistical results of a pilot study wherein the patient distribution in the number of nasal bleeding after NTI was used as a standard (E group, n = 10; T group, n = 10). As the use of statistical tests in the absence of reliable sample size calculation decreases its weightage, we calculated our final sample size considering an expected dropout rate of 0.05 based on our pilot study. Hence, if a dropout rate (R) is expected, a simple but adequate adjustment is provided by N_d_ = N/(1 − R)^2^, where N is the sample size calculated assuming no dropout and N_d_ is the estimated sample size required when dropouts are expected [18]. Therefore, after adjusting for dropouts, a final sample of 197 patients was observed. For statistical testing, Chi-Square test of independence was used to test for a relationship between two categorical variables, and the Mann–Whitney U test was used to compare differences between two independent groups for continuous variables. Wilcoxon signed-rank test was used to compare differences between two dependent groups for continuous variables. The two-sided statistical significance level was set at *p* ≤ 0.05. Statistical analysis of recorded data were performed using IBM® SPSS® Statiscs Ver26.

## Results

From October 2019 to September 2020, 206 patients were selected to participate in this study. The participant CONSORT flow diagram is presented in Fig. 1. From the 197 patients randomly assigned into two groups on the basis of the vasoconstriction method used (E or T), 5 patients dropped out during the trial. The final analytical sample included 192 patients (96 per group).

Demographic characteristics, operation and anesthesia time, and the years of experience of the intubator are presented in Table 1.

**Table 1:** Patient’s demographic characteristics and NTI related information

	Group E	Group T	*P* value
Male/Female	38/58	41/55	0.65
Age (years)	32 (25–44)	30 (25–46)	0.89
Hight (cm)	162 (156–169)	164 (158–170)	0.31
Weight (kg)	55 (49–61)	57 (50–68)	0.11
Operation time (min)	75 (47–125)	90 (69–149)	0.08
Anesthesia time (min)	127 (91–185)	136 (103–206)	0.18
Experience of intubator (years)	4 (1–6)	4 (1–6.25)	0.98

Values are n or median (quartile1-quartile3)

No statistical differences between the groups were observed. The presence of bleeding was observed in 12 patients from the E group and in 14 patients from the T group, representing a similar proportion in the two groups (12.5% vs.14.5%, *P* = 0.63). None of the patients had severe bleeding. No significant difference between the groups (*P* = 0.78) regarding the degree of bleeding was observed (Table 2).

**Table 2:** Patient’s distribution of degree of bleeding

	Group E	Group T	*P* value
None	84	82	0.78
Mild	10	10	
Moderate	2	4	
Severe	0	0	

Values are n

Table 3 shows the change in hemodynamics with the use of vasoconstrictors. In group E, the MAP significantly increased after the vasoconstrictor was applied (before 71.5 mmHg vs. after 80.0 mmHg, *P* = 0.001), whereas no significant differences were observed in the HR (before 64.5 beat/min vs. after 65.0 beat/min *P* = 0.24). Likewise, in group T, the MAP significantly increased after the vasoconstrictor was applied (before 72 mmHg vs. after 77 mmHg, *P* = 0.04). In contrast, the HR remained stable during the NTI procedure (before 68.5 beat/min vs. after 68.0 beat/min, *P* = 0.91).

**Table 3:** Hemodynamics before and after the use of vasoconstrictors

	Before	After	*P* value
MAP (mmHg) group E	71.5 (72–90)	80 (66–82.5)	0.001
HR (beat/min) group E	64.5 (58–72)	65 (60–79)	0.24
MAP (mmHg) group T	72 (63–82.5)	77 (69–87.5)	0.04
HR (beat/min) group T	68.5 (57.75–78)	68 (58.75–79)	0.91

Values are median (quartile1–quartile3)

*MAP* Mean atrial presure, *HR* Heart rate

No arrhythmias induced by vasoconstrictor use were observed in the two groups.

## Discussion

In this study, using E or T as a vasoconstrictor, we observed no difference in the frequency and degree of bleeding between the groups. Both T and E are excellent drugs to prevent bleeding because there was no severe bleeding in either group.

Nasal bleeding due to NTI is mostly caused by damaging of the venous plexus in the middle nasal mucosa, the curvature of the nasal septum, and adenoid enlargement [19]. In addition, nasal bleeding during NTI can obstruct the airway patency and cause serious complications such as airway obstruction. Considering that persistent bleeding can also affect the operative field, every effort should be made to prevent bleeding whenever possible.

Several pretreatment methods for nasal intubation using vasoconstrictors [19], nasal airway tube [20], or cotton swab [10] have been reported. Generally, cotton swab is used after the use of vasoconstrictors or nasal airway tube to check the passage of NTI. As for the nasal airway tube, it is reported that inserting a nasal airway into the nostril just before the NTI may facilitate the intubation process by preparing the nasal passage [20]. However, in this study, we did not use nasal airway or cotton swab in order to examine the effects of vasoconstrictors alone.

Vasoconstrictors are used to achieve local hemostasis by constricting the intramucosal capillaries and reducing blood flow, thereby expanding the nasal volume and facilitating the passage of the intubation tube through the nasal cavity [19]. E is the most common vasoconstrictor for local hemostatic purposes, with recommended concentrations of 0.1–0.02% [21]. Previous studies [11, 22] using E to prevent nasal bleeding reported hemorrhage rates of 37% [11] and 60% [22], which are considerably higher than the 12.5% of nasal bleeding observed in the present study. This discrepant finding may be attributed to the concentration of E, nasal stenosis, and left-right differences in the nasal cavity used. Note that both previous studies [11, 22] used an E concentration of 0.01%, whereas in our study we used a concentration of 0.02%. Although some blood pressure fluctuation was observed before and after use of E, it was not clinically problematic, and the 0.02% concentration could be used without problems. Furthermore, E has an onset of action of 5 min and lasts for 1 h [22]. For practical reasons, we set a time of 2 min (instead of 5 min) and we found that a 2-min period was sufficient to achieve hemostasis.

In this study, as patients were undergoing oral surgery, a computed tomography of the facial area was taken in all cases prior to the study. Therefore, it was possible to include nasal stenosis as an exclusion criterion for the current study. Hence, despite the 4-year average intubation experience, the passage of the intubation tube was smooth and performed at once in all patients. Moreover, all patients were intubated using the right nasal cavity, as we have previously reported less epistaxis in the right nasal cavity compared with the left nasal cavity [7]. Unlike the current study, Sonan et al. [22] used the left nasal cavity for intubation, but it is unclear which nasal cavity was used in the study by Jaegyok et al. [11] We believe that the above three factors contributed to the lower percentage of bleeding observed in our study than in the previous studies [11, 22].

In contrast, we also used T as a vasoconstrictor in this study. There are reports that nasal T infusion improves rhinitis [23] and sleep apnea [24]. To the best of our knowledge, there are no reports regarding use of T for preventing nasal bleeding during NTI. Our findings indicate that T is as effective as E regarding prevention of nasal bleeding during NTI. The blood pressure fluctuation observed in the present study during the use of both drugs is due to the sympathetic stimulating effects of both drugs. Although the blood pressure fluctuations in both groups were clinically safe, E increased blood pressure more than T, suggesting that E stimulates alpha receptors more strongly than T within the concentrations used in this study. Although patients with hypertension were excluded from the study, the use of both drugs increased blood pressure of normotensive patients, suggesting the need for extremely careful judgment while using these drugs in hypertensive patients.

In the present study, we also found that the use of both drugs did not induce arrhythmias. It has also been reported that the use of E for the prevention of nasal bleeding usually has a minimal effect on circulation in many patients [25, 26]. However, sudden tachycardia was reported when E was used to prevent nasal bleeding [16]. In that study, it was assumed that severe tachycardia was triggered by submucosal migration of an epinephrine-soaked swab. Hence, even at normal concentrations of E, when the mucous membrane is injured by swabs or other means, it is important to consider that a rapid increase of concentration of E in the blood may induce arrhythmia. In the present study, total intravenous anesthesia was used in all patients. However, previous studies reported a decrease in the arrhythmia range of E in combination with volatile anesthetics with halogenated anesthetics ranging from halothane to sevoflurane [27, 28]. Therefore, attention should be given to variations in circulation dynamics while using E. Another drug that is less likely to cause circulatory changes and arrhythmias is the alpha-2 agonist dexmedetomidine. Sukegawa et.al reported that locally injected dexmedetomidine at a relatively high concentration inhibits local acute inflammatory responses, including edema, the accumulation of leukocytes [29]. In the future, dexmedetomidine may be applied to the prevention of nasal bleeding during NTI.

The first limitation of this study is the exclusion of patients with nasal stenosis; different results could be expected if patients with nasal stenosis were included. However, mixing of patients with and without nasal stenosis in the same trial must be avoided. This issue should be investigated in future studies. Secondly, this study does not have a control group that uses saline instead of E or T. If obvious nasal bleeding is observed in the control group, urgent use of E can resolve it and the presence of a control group may have further emphasized the importance of the results of this study using E or T. However, despite the availability of drugs to reduce nasal bleeding during NTI, not using them was rejected by the ethics committee at the review stage. Therefore, we are unable to conduct any further experiments.

## Conclusion

In conclusion, nasal treatment with E or T shows no difference in nasal bleeding during NTI. Although no arrhythmia associated with E was observed in this study, it has been reported in the literature. Therefore, as the frequency and degree of nasal bleeding were comparable, nasal treatment with T could reduce the risk of NTI.

## Data Availability

The datasets analyzed during the current study are available from the corresponding author on reasonable request.

## Abbreviations

NTI: Nasotracheal intubation
E: Epinephrine
T: Tramazoline
MAP: Mean arterial pressure
HR: Heart rate
